# Exploring women’s knowledge and their perception of risk of uterine cancer in Lagos, Nigeria: a multi-facility based cross-sectional study

**DOI:** 10.3332/ecancer.2025.1919

**Published:** 2025-05-30

**Authors:** Adeyemi Adebola Okunowo, Rukayat Omolola Salawu-Giwa, Oluwaseun Emmanuel Familusi, Salimat Abisoye Yusuf-Awesu, Fadekemi Ooreoluwapo Gabriel-Raji

**Affiliations:** 1 Department of Obstetrics and Gynaecology, College of Medicine, University of Lagos (CMUL), PMB 12003, Idi-Araba, Lagos, Nigeria; 2 Department of Obstetrics and Gynaecology, Lagos University Teaching Hospital (LUTH), PMB 12003, Idi-Araba, Lagos, Nigeria; 3 Queen’s Medical Centre, Nottingham University Hospitals NHS Trust, Derby Road, NG7 2UH Nottingham, UK; a https://orcid.org/0000-0002-8375-4443

**Keywords:** uterine cancer, endometrial cancer, knowledge, symptoms, risk factors, risk-reducing measures, Lagos, Nigeria

## Abstract

**Introduction:**

The absence of an established screening strategy to effectively detect uterine cancer (UC) at its early stage and the favourable outcome associated with early disease has put a premium on the need for increased public knowledge of UC to raise awareness about the risk of the disease and encourage the prompt presentation of suspicious symptoms and early diagnosis to improve health outcomes and survival.

**Objectives:**

We, therefore, sought to explore the knowledge of UC symptoms, risk factors, risk-reducing health measures and their perceived risk of developing the disease among women in Lagos, Nigeria.

**Methods:**

The study was a descriptive cross-sectional study conducted among 555 community women who attended government-owned secondary health facilities in three randomly selected Local Government Areas in Lagos, Nigeria. Information on sociodemographic and reproductive characteristics, awareness and knowledge about UC, its symptoms, risk factors, risk-reducing health measure, and perception of the risk of having UC were collected using an interviewer-administered questionnaire to assess women’s knowledge and their perception of risk of UC. Data analysis was done using SPSS version 23.

**Results:**

Though 58.4% of the respondents were aware of UC, only 27.4%, 34.9% and 39.3% had good knowledge of the risk factors, symptoms and risk-reducing health measures of UC, respectively. The overall knowledge about UC was low with 25.0% having good knowledge about UC, while only 11.2% believed they may be at risk of developing UC. Being 25 years and below in age [adjusted odd ratio (AOR) = 2.55, CI = 1.36–4.77, *p* = 0.003], having at least a secondary education (AOR = 1.67, CI = 1.06–2.91, *p* = 0.046), being unmarried (AOR = 2.69, CI = 1.39–5.21, *p* = 0.003), a Christian (AOR = 1.89, CI = 1.09–3.27, *p* = 0.023), knowing someone with UC (AOR = 6.62, CI = 3.12–14.01, *p* < 0.001) and discussion with a doctor about UC (AOR = 5.72, CI = 3.43–9.53, *p* < 0.001) significantly predicted good knowledge of UC. Similarly, being 25 years and below in age (AOR = 2.49, CI = 1.20–5.17, *p* = 0.014), being a Muslim (AOR = 3.08, CI = 1.58–5.99, *p* = 0.001), knowing someone with UC (AOR = 3.11, CI = 1.27–7.57, *p* = 0.013) and having good knowledge of UC (COR = 5.88, CI = 2.80–12.35, *p* < 0.001) significantly influenced perception of the risk of developing UC.

**Conclusion:**

Women’s knowledge of UC and their perceived risk of developing the disease is very low in Lagos, Nigeria. Age, education, marital status, religion, knowing someone with UC and discussion with a doctor significantly influenced their knowledge and perceived susceptibility to the disease. There is a need for strategic educational interventions to address the knowledge gaps to improve health outcomes.

## Introduction

Cancer of the corpus uteri, also known as uterine cancer (UC) and commonly called cancer of the womb comprises all cancers that affect the uterus, with endometrial cancer being the most common type. According to the latest GLOBOCAN 2022 global cancer statistics, cancer of the corpus uteri is the sixth most common cancer in women and the 2nd most common gynaecological cancer worldwide after cervical cancer [1]. In Nigeria, it is the third leading gynaecological cancer after cervical and ovarian cancer, accounting for 1,627 new cases and 543 cancer-related deaths in 2022 [2].

UC can arise from any of the tissue components that make up the uterus, usually from the endometrial lining leading to endometrial cancer (EC), and from the myometrium and connective tissue resulting in sarcoma. EC is the most common type, accounting for approximately 90% of all uterine malignancies [3]. EC is the most common gynaecological malignancy in many high and middle-income countries including the United States and the United Kingdom (UK) with increasing incidence and a concomitant increase in its mortality rate [3–6]. Traditionally, EC is a disease of white women of European ancestry but this has changed over the past few decades with black women of African ancestry now disproportionately affected by EC and its aggressive subtypes with resultant poorer outcomes and increased mortality [3, 5]. It has been reported that black women have at least a 2-fold higher risk of developing high-grade EC compared to Whites and Hispanics [3, 7, 8]. Similarly, a 2-fold increase in mortality has been reported among black women compared to their White counterparts (10.0 per 100,000 versus 4.8 per 100,00) [9].

Likewise, there has been an increasing trajectory in the trend of EC in many low- and middle-income countries (LMICs) including Africa [4, 10]. Based on this trajectory, EC is projected to become the second most common gynaecological malignancy in Africa from its current third position in line with the rising global trend [10]. Studies in Nigeria, have also reported an increasing trend in the incidence of EC, despite its position as the third most common gynaecological cancer in most parts of the country [11–14].

Unlike cervical cancer, UC has no established screening strategy to aid early detection. In the absence of this, early presentation and diagnosis are essential to improve the outcomes and survival of women with UC as early-stage disease is associated with a high cure rate, better prognosis and survival [15]. To achieve this, there is a need to invest in public awareness and health education of women about the disease, the benefit of early presentation, diagnosis and treatment. This public health intervention should strategically target the identified areas of poor knowledge and misunderstandings about UC and aim to increase public awareness about the disease’s symptoms, danger signs, risk factors and how to reduce the risks of developing the disease. Very few studies predominantly in high-income countries (HICs) have examined women’s knowledge about UC. These studies have reported poor public awareness and knowledge of the disease, its symptoms and risk factor, even among women in HICs [5, 15–17]. A 51.5% UC awareness rate was reported in Germany [18], while an abysmal rate of 6% was reported among rural women in Australia [19]. In the UK, only 24% and 13% of women could correctly identify at least two symptoms and risk factors for UC, respectively [16]. Among the few studies conducted in LMICs, 57.0% of educated women who utilise digital media platforms in a Southwestern state in Nigeria were aware of UC, while 46.0% knew the common symptoms of UC [20]. Another study in Egypt among elderly women with postmenopausal bleeding showed that all the study participants had a low level of awareness of UC and poor knowledge of its symptoms and risk factors [21].

Many women normalise key symptoms of UC as normal age-related symptoms or attribute them to benign conditions, preventing early presentation at the hospital [5, 15, 16, 22]. As a result, the suspicion of cancer-related symptoms is usually disregarded by women due to a lack of adequate knowledge about the cancer symptoms [5, 22]. Similarly, knowledge about the risk factors of UC especially that of obesity is poor and obesity is a prevalent condition and a major risk factor for UC [17, 23]. Lack of knowledge about this fact could affect women’s attitude and preventive practices toward obesity and obese women’s perception of their risk of UC and early health-seeking behaviour. It is believed that a third of UC especially EC can be prevented by controlling obesity which is a preventable and modifiable condition, educating the population about other non-modifiable risk factors and encouraging positive health behaviours [16, 17, 23]. In addition, early identification of women at risk of UC and appropriate counseling on risk modification and danger signs of UC will result in improved health outcomes and survival [24].

It is, therefore, imperative that women are not just aware and knowledgeable about UC, but also appropriately understand their risk of developing UC and that being a woman itself predisposes them to the risk of UC. Appropriate understanding of UC especially its risk factors and symptoms can aid women’s self-assessment of their risk of UC, positive healthcare practices and encourage early presentation leading to early diagnosis, prompt treatment and improved health outcomes.

Lagos, being a cosmopolitan and metropolitan city, and the most populous state in Nigeria has a strategic population of women at high risk of UC. To the best of our knowledge, there is no identifiable published data on women’s knowledge of UC and their perceived risk of developing the disease in Lagos, Nigeria. We, therefore, sought to explore the knowledge of UC symptoms, risk factors, risk-reducing health measures and their perceived risk of developing the disease among women in Lagos, Nigeria.

## Methodology

### Study design and setting

This descriptive cross-sectional study was conducted among community women who attended government-owned secondary healthcare facilities in Lagos state, Nigeria over a 3 month period between 1 March and 31 May 2018. Lagos state is located in the Southwestern part of Nigeria. It is the smallest state in Nigeria, yet the most populous state in Nigeria with an estimated population of about 24.6 million inhabitants in 2015. It has 5 administrative divisions and 20 local government areas (LGAs) [25]. Each LGA has at least one government-owned secondary healthcare facility, called a general hospital, which provides a secondary level of care to the people living within the communities in the LGA.

### Study population

This comprised community women who visited the gynaecological clinics of the government-owned secondary healthcare facilities in their respective LGAs during the study period.

### Eligibility criteria

All women who visited the gynaecological out-patient clinics of the selected government-owned secondary healthcare facilities, who are 18 years and above and who gave consent to participate in the study were recruited and enrolled into the study. However, women below the age of 18 years, with a personal history of UC and who are not willing to participate in the study were excluded from the study.

### Sample size determination

The sample size was calculated using the appropriate formula (*n* = *Z*^2^ *p* (1 − *p*)/*d*^2^) [26] with type 1 error of 5% (*Z* = 1.96), an absolute error margin of 5% (*d* = 0.05) and proportion of women aware of risk factors of EC (*p*) of 37.0% [19]. The calculated minimum sample size was 358; after adjusting for a non-response rate of 15%, the final sample size was 412.

### Sampling technique

A multistage sampling technique was used to recruit study respondents in the following stages:

#### Stage 1: Selection of Local Government and General Hospitals

Three LGAs were selected from the state’s 20 LGAs by a simple random sampling method using the ballot technique. Numbers were allocated to each of the different LGAs for identification purposes. These numbers were written on ballot papers, folded and put into a ballot box. Three ballot papers were randomly picked consecutively after thoroughly mixing the documents in the box. The numbers on the randomly picked ballot papers represented Oshodi–Isolo, Agege and Mushin LGAs. The General Hospitals within these randomly picked LGAs were selected for the study. These included Mushin General Hospital, Isolo General Hospital and Orile–Agege General Hospital in Mushin, Oshodi–Isolo and Agege LGAs, respectively.

#### Stage 2: Selection of respondents

Equal numbers of respondents were selected from each General Hospital to allow for equal representation of women from the LGAs. The respondents were recruited from the gynaecological outpatient clinic using a systematic random sampling method to minimise selection bias. Every third patient queued to see the doctor was recruited after randomly selecting the first respondent. A total of 175 female respondents were enrolled and interviewed at each General Hospital.

### Survey tool

A structured questionnaire (see Supplementary material) was designed to collect the necessary information from the respondents. The content of the structured questionnaire was adapted from the tools used in previous studies that assessed knowledge of UC and from an extensive literature search on the symptoms, risk factors and risk-reducing health measures of UC. The content of the questionnaire was further reviewed by experts in the field of gynaecological oncology and appropriate revisions were made to the questionnaire based on the feedback from the experts. Thereafter, the questionnaire was piloted among a convenient sample of 20 women who visited the gynaecological outpatient clinic at the Lagos University Teaching Hospital to assess its usability, appropriateness of its content to the target population, clarity of its instructions and its effectiveness in fulfilling its objective. The outcome of the pilot study was used to improve the quality of the questionnaire.

The content included information on sociodemographic characteristics, reproductive characteristics, awareness about UC, knowledge about symptoms, risk factors, and risk-reducing health measures of UC, perception about risk of developing UC, knowledge of someone with UC and prior discussion with a doctor/nurse about UC. The sociodemographic characteristics include age, marital status, education level, ethnicity, religion and occupation. The Occupation was categorised as skilled, semi-skilled, unskilled and unemployed. Skilled occupations require a tertiary level of education, specialised training or certifications such as doctors, engineers, lawyers, nurses, managers, teachers, accountants, consultants and other professional jobs. Semi-skilled occupations require a secondary level of education and a partial skill set that does not require advanced training or certification such as technicians, clerks, secretaries, salesmen/saleswomen, office workers, artisans and so on. Unskilled occupations may or may not require elementary education and do not require any specific skill set, e.g., security guard, housekeeping, petty trading, fast food attendants, waiters and so on [27] The unemployed are those who are currently not engaged in productive economic activities such as housewives, retirees and so on. Reproductive characteristics included the total number of deliveries, number of children alive and the presence or absence of menopause. A woman was considered to be postmenopausal if she has stopped menstruating for at least 1 year. On the other hand, participants who are still menstruating regularly or who have not yet stopped menstruation for up to 1 year were considered to be pre-menopausal.

The questionnaire was interviewer-administered to improve the quality of the data collected. To overcome the inherent biases in the interviewer-administered survey used in our study, the interviewers were unknown to the respondents and trained to have an in-depth understanding of the content of the study questionnaire, how to present the questions with clarity in a neutral and professional manner without showing an unconscious preference or aversion for any response and record the exact responses of the respondents accurately without undue personal interpretations. The interviewers were also trained on the need to be courteous, clearly introduce the purpose of the study to the respondents, inform respondents about the voluntariness of the study, the importance of providing the answers that apply to them as there are no ‘right’ or ‘wrong’ answers to the questions and that their responses would be treated with much confidentiality and respect. The respondents were also informed that no identifying information would be collected nor would their identity be linked to their responses. Strategies such as the use of recent events to aid the recall of previous events, and the use of memory aids such as probes, linkage to key events or activities that are traceable to the event to be recalled were used to mitigate recall bias.

### Assessment of knowledge and risk perception of uterine cancer

A scoring system used in previous studies [28, 29] that assessed cancer knowledge was adopted and modified to assess UC knowledge. Each correctly identified UC symptom, risk factors and risk-reducing health measures was allotted a score of 1, an incorrect or ‘I don’t know’ response was allotted a score of zero and the total score was calculated for each respondent. The maximum score for UC symptoms and risk factors was 12, respectively. Respondents with a score of 6 and above were classified as having ‘good knowledge’ of UC symptoms and risk factors, respectively. In contrast, respondents with scores between 1 and 5 were classified as having ‘poor knowledge’, while respondents with a score of 0 were classified as having ‘no knowledge’. The maximum score for knowledge of risk-reducing health measures was 7. Respondents with a score of 4 and above were classified as having ‘good knowledge’ of risk-reducing health measures. In contrast, respondents with scores between 1 and 3 were classified as having ‘poor knowledge’. Respondents with a score of 0 were regarded as having ‘no knowledge.’

The overall knowledge of UC was determined by calculating the maximum cumulative score for knowledge of symptoms, risk factors and risk-reducing health measures, which was 31. Respondents with a score of 15 and above were classified as having ‘good knowledge’ of UC. In contrast, women with scores between 1 and 14 and 0 were classified as having ‘poor knowledge’ and ‘no knowledge’ respectively. The perception of the risk of developing UC was assessed by asking respondents, ‘Do you think you are at risk of having uterine cancer?’ Respondents who said ‘yes’ were considered to be aware of their risk of developing UC, while those who said ‘no’ or ‘I don’t know’ were categorized as being unaware of the risk of developing UC.

### Data analysis

The data was analysed using Statistical Package for Social Sciences version 23.0, IBM Corp., Armonk, NY, USA, after it was cleaned and validated. Numerical data was checked for normality using Shapiro–Wilk’s test and normally distributed variables were expressed as mean ± standard deviation while skewed variables were presented as the median and interquartile range.

Explanatory and dependent variables were stratified and grouped into categories for analysis and descriptive statistics were generated and presented as frequencies and percentages in tables and charts. Bivariate analysis was done using Pearson’s chi-square test or Fischer’s exact test as appropriate (when the expected cell value was less than 5) to compare grouped variables. Univariable regression analysis was done and crude odd ratios generated. Further multivariable regression analysis was conducted using a stepwise backward elimination technique for variables with *p*-value < 0.2. The level of statistical significance was set at a *p*-value < 0.05 and 95% confidence interval.

## Result

507 respondents out of a total of 555 participants had complete data and were included in the final analysis giving a response rate of 91.4%.

### Characteristics of the study participants

Table 1 shows the characteristics of the study participants. The mean age of the participants was 32.9 ± 8.7 years [95% Confidence interval (CI): 32.2–33.7 years] with the majority (44.6%, 226) between the ages of 30 and 39 years. The majority of the participants were unemployed (31.4%, 159), married (74.4%, 377), of the Yoruba ethnicity (60.6%, 307), practiced Christianity (66.9%, 339) and had a secondary level of education (39.1%, 196). The median parity was 1 (0–3) [95% CI: 0–1] and most of the participants were pre-menopausal (88.2%, 447). Only 8.7% (44) knew someone who had UC and 23.3% (118) of the respondents had had a form of discussion with a doctor about UC.

### Awareness and knowledge of uterine cancer

**Figure 1.**
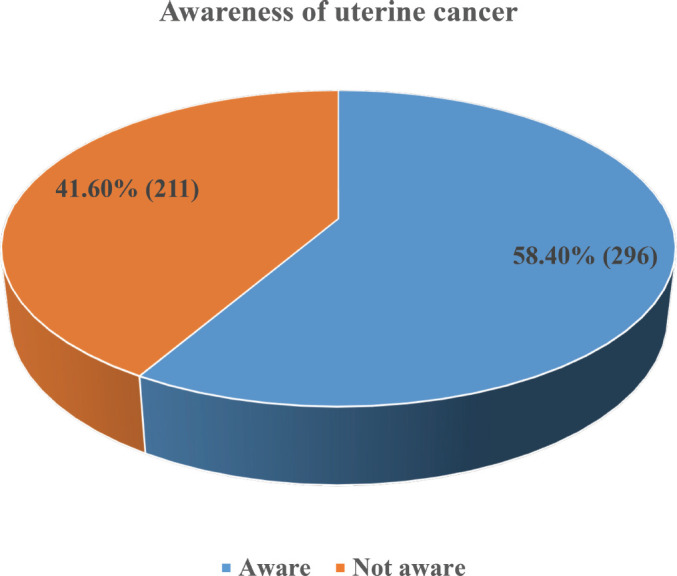
Awareness of uterine cancer. Majority (58.4%, 296) of the respondents have heard of uterine cancer before.

**Figure 2.**
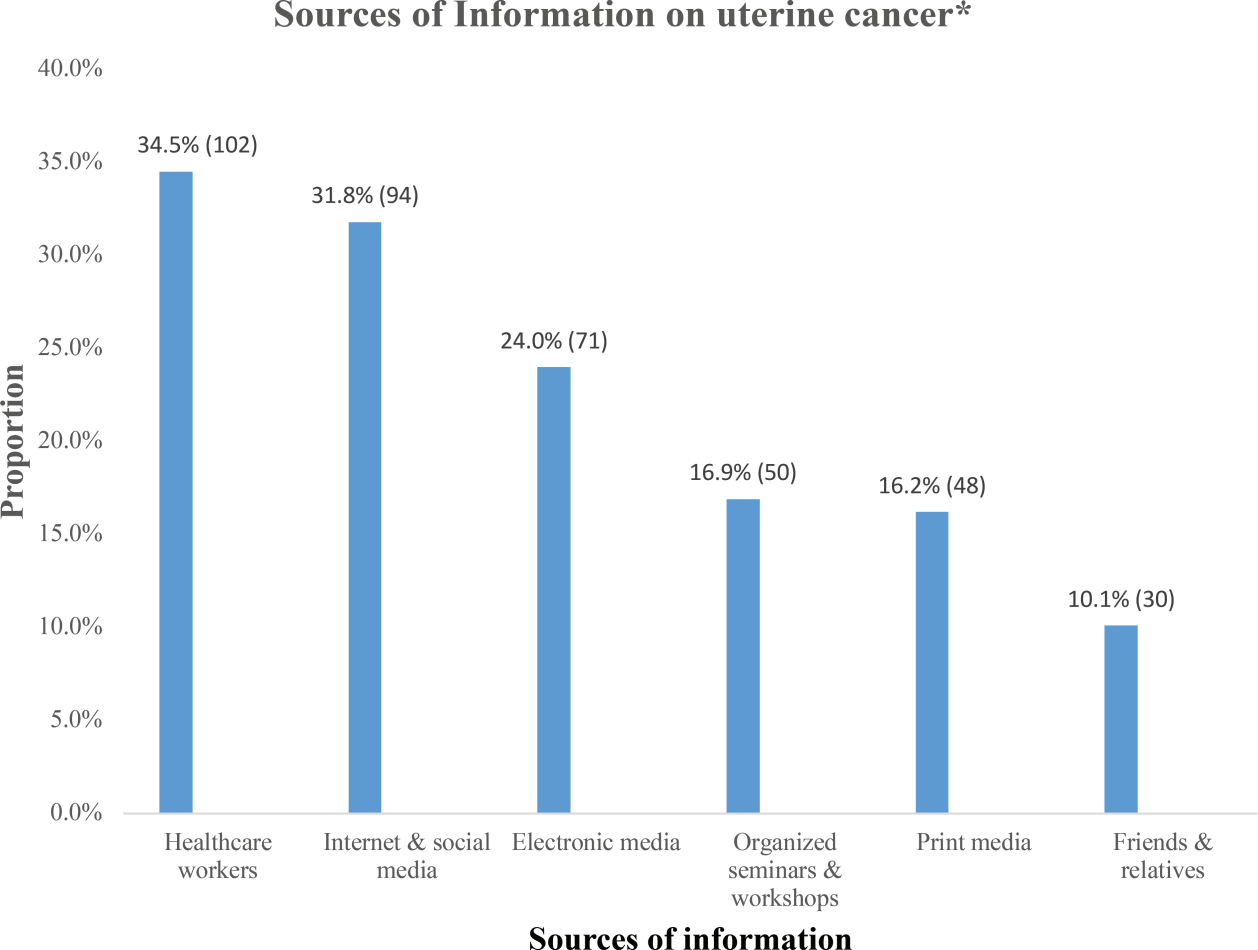
Respondents’ sources of information on uterine cancer. *Multiple responses observed (n = 296) Healthcare workers (34.5%, 102), internet and social media (31.8%, 94) and electronic media (24.0%, 71) were the leading sources of information on uterine cancer among the respondents.

More than half (58.4%, 296) of the respondents had heard about UC and were aware of it (Figure 1). The main sources of information about UC were healthcare workers (34.5%, 102), the internet and social media (31.8%, 94), electronic media (24.0%, 71) and organised workshops and seminars (16.9%, 50) (Figure 2).

Table 2 shows the symptoms of UC identified by the participants. Vaginal bleeding after menopause (27.4%, 139), lower abdominal pain (25.2%, 128), heavy or prolonged uterine bleeding (24.7%. 125) and unexplained weight loss (24.1%, 122) were the most commonly identified symptoms of UC among the participants. On the other hand, abdominal swelling (17.9%. 91), constipation (18.1%, 92) and loss of appetite (97, 19.1%) were the least identified symptoms associated with UC.

The most commonly identified risk factors were family history of UC (29.2%, 148), advanced age (23.3%, 118), use of unopposed estrogen hormone drugs (23.3%, 118) and obesity (22.7%, 115). The use of tamoxifen (13.0%, 66), the presence of hypertension (13.2%, 67), diabetes mellitus (14.0%, 71) and lack of child delivery (16.0%, 81) were the least identified risk factors of UC (Table 3).

Practice of eating a healthy diet (52.3%, 265), regular medical check-up reviews with the gynaecologists especially in the presence of risk factors (50.9%, 258), maintaining a healthy weight (50.3%, 255) and engaging in regular exercises and physical activities (48.3%, 245) were the most common risk-reducing health measures identified. The use of progesterone-containing intrauterine devices (17.8%, 90), birth control pills (20.9%, 106) and avoidance of estrogen-only medications (32.3%, 164) were the least identified risk-reducing health measures (Table 4).

**Figure 3.**
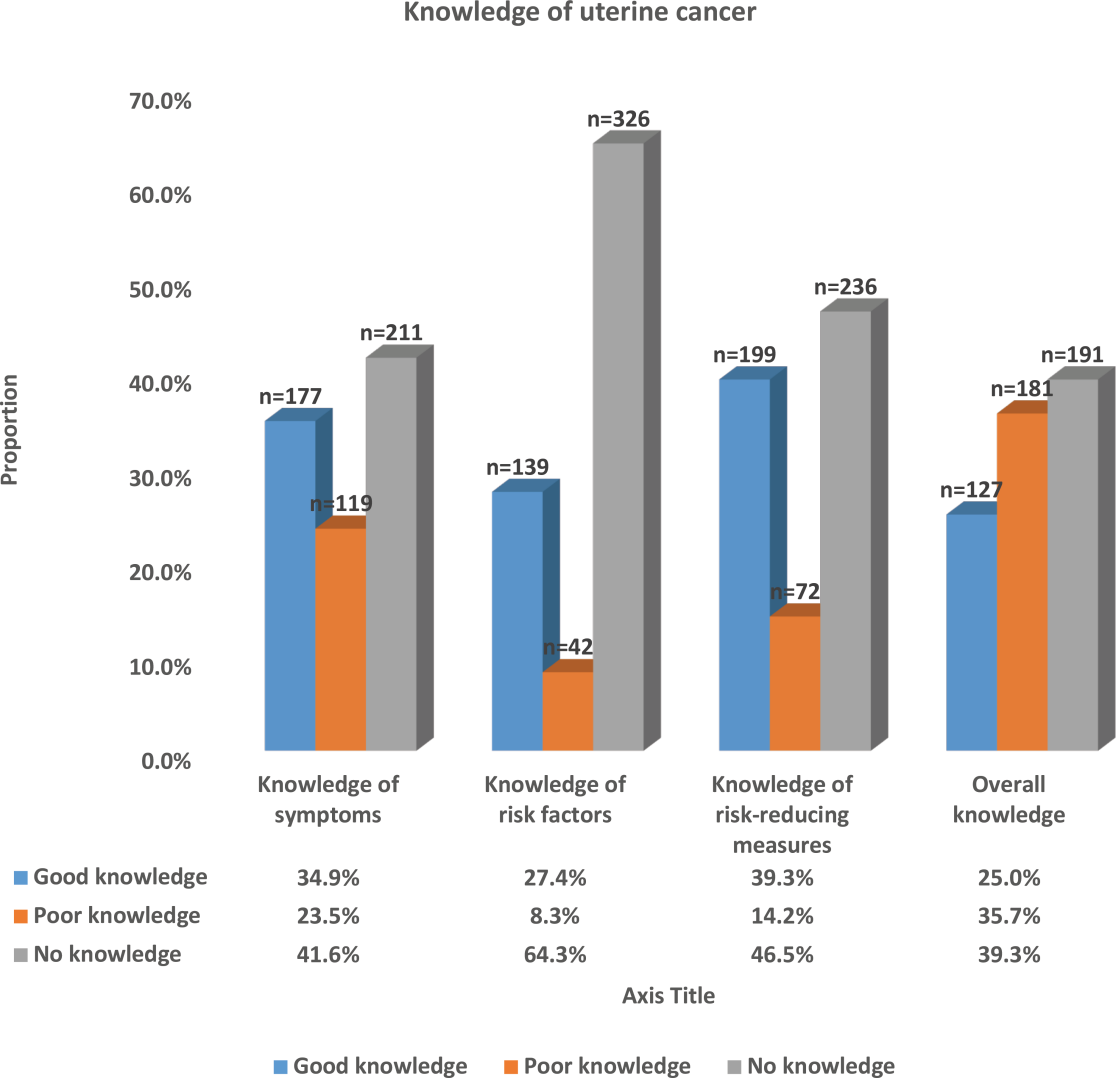
Distribution of knowledge of uterine cancer among respondents. Most of the respondents lack complete knowledge about either the symptoms (41.6%, 211), risk factors (64.3%, 326) or risk-reducing measures (46.5%, 236) of uterine cancer. Only 25.0% (127) had good overall knowledge of uterine cancer, while others (75.0%) had either poor or no knowledge

Figure 3 shows the pattern of the knowledge of UC observed among the respondents. The majority (41.6%, 211) of the participants had no knowledge of the symptoms of UC, approximately a third (34.9%, 177) had good knowledge, while the remaining 23.5% (119) had poor knowledge of UC symptoms with a mean knowledge of UC symptom score of 3.62 (95% CI: 3.23–4.02). Most of the participants demonstrated a complete lack of knowledge of the risk factors of UC with more than two-thirds (64.3%, 326) having no knowledge of the risk factors, while only about a quarter (27.4%, 139) had good knowledge of the risk factors. The mean score of the knowledge of UC risk factors was 2.81 (95% CI: 2.42–3.17). Knowledge of measures that reduce the risk of UC was also low among the participants. Less than two-fifths (39.3%, 199) of the participants had good knowledge of these measures, while the majority (46.5%, 236) had no knowledge of these measures. The mean knowledge score of risk-reducing measures of UC among the respondents was 2.49 (95% CI: 2.23–2.73). Only a quarter of the participants (25.0%, 127) had an overall good knowledge of UC, while the majority (39.3%, 191) had no knowledge about it and 35.7% (181) had poor knowledge. The overall mean knowledge score of UC was 8.92 (95% CI: 8.05–9.79).

### Factors influencing knowledge of uterine cancer

Tables 5 and 6 depict the socio-demographic, reproductive and relational factors influencing the knowledge of UC among the participants. Participants’ age, marital status and religion significantly influenced their knowledge of UC (*p* = 0.035, <0.001 and 0.028, respectively). Participants between the age of 20 and 29 years had better knowledge of UC compared to other age strata, while women between 40 and 49 years and ≥50 years were the least with had good knowledge of UC (*p* = 0.035). More than half (60.6%, 77) of the participants with good knowledge were married women (*p* < 0.001), while approximately three-quarters (74.8%, 95) were Christians (*p* = 0.028). Though the knowledge of UC increased across the educational strata with women who had tertiary education having a higher level of knowledge of UC and women without school education having the least level of knowledge, this was not statistically significant (*p* = 0.086). The number of deliveries and children significantly influenced the knowledge of UC (*p* = 0.013 and 0.001, respectively), with women with a low number of deliveries and children accounting for the higher proportion of women with good knowledge of UC compared to those with a higher number of deliveries and children. Similarly knowing someone with UC and having a previous discussion with a doctor about UC were significantly associated with having good knowledge compared to their counterparts (*p* = <0.001, respectively).

To further understand the level of association between these factors and UC knowledge, and to determine the factors that predict good knowledge of UC, univariate and multivariate regression analyses were done as shown in Table 7. Being 25 years and below in age [crude odd ratio (COR) = 2.92, CI = 1.83–4.66, *p* = <0.001], having at least a secondary education (COR = 1.57, CI = 1.18–2.52, *p* = 0.019), being nulliparous (COR = 1.56, CI = 1.04–2.34, *p* = 0.033), unmarried (COR = 2.64, CI = 1.71–4.08, *p* < 0.001), a Christian (COR = 1.66, CI = 1.05–2.60, *p* = 0.029), knowing someone with UC (COR = 6.43, CI = 3.35–12.36, *p* < 0.001) and prior discussion with a doctor about uterine cancer (COR = 6.47, CI = 4.11–10.18, *p* < 0.001) were significantly associated with increased odds of having good knowledge of UC on univariate analysis. On multivariate analysis, the independent predictors of good knowledge of UC were being 25 years and below in age [adjusted odd ratio (AOR) = 2.55, CI = 1.36–4.77, *p* = 0.003], having at least a secondary education (AOR = 1.67, CI = 1.06–2.91, *p* = 0.046), being unmarried (AOR = 2.69, CI = 1.39–5.21, *p* = 0.003), a Christian (AOR = 1.89, CI = 1.09–3.27, *p* = 0.023), knowing someone with UC (AOR = 6.62, CI = 3.12–14.01, *p* < 0.001) and prior discussion with a doctor about UC (AOR = 5.72, CI = 3.43–9.53, *p* < 0.001).

### Perception of the risk of developing uterine cancer and factors influencing it

**Figure 4.**
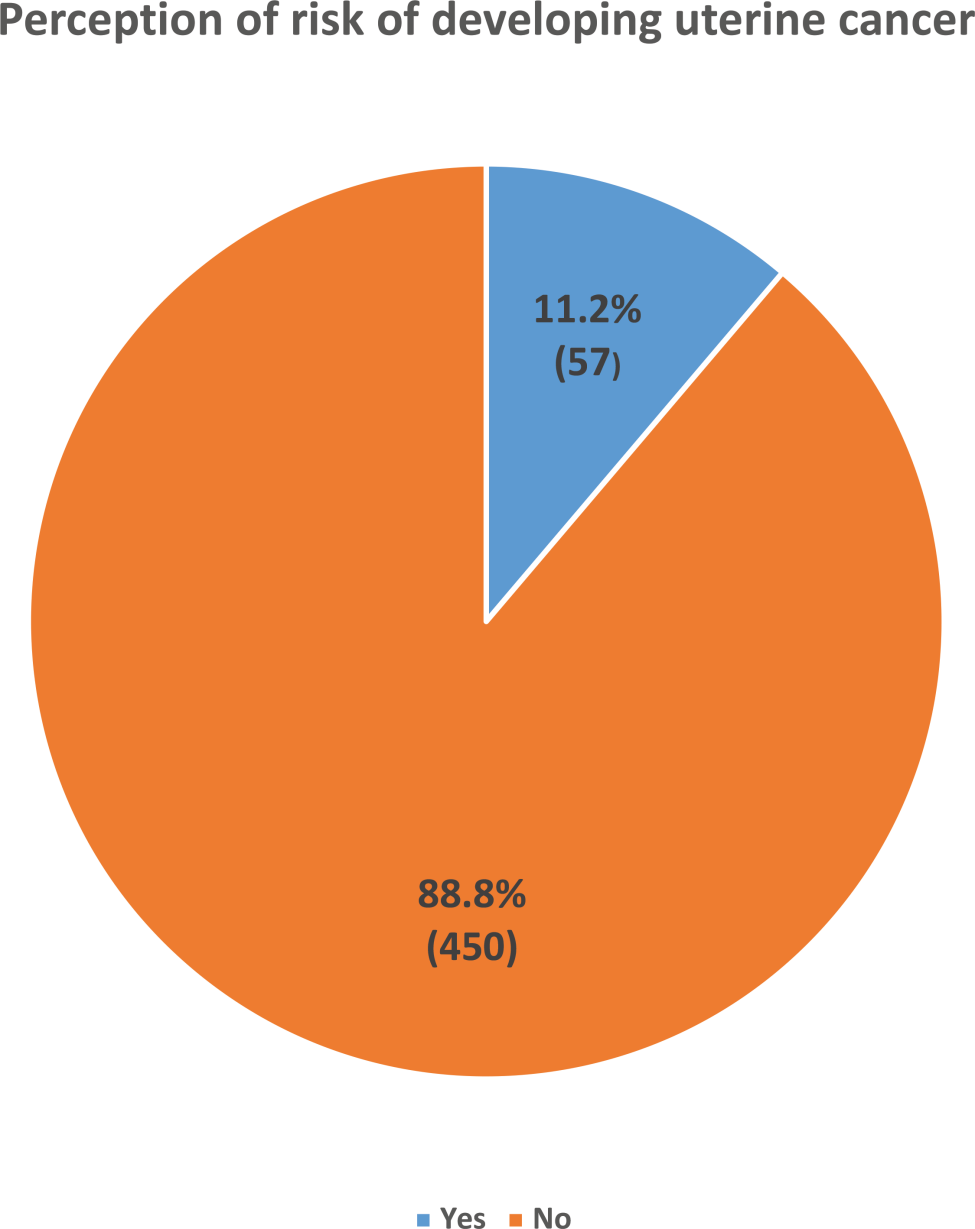
Showing the perception of the risk of developing uterine cancer among the respondents. Majority (88.8%, 450) of the respondents believed they were not at risk of developing uterine cancer.

Only 11.2% (57) of the study participants perceived that they may be at risk of developing UC, while the majority (88.8%, 450) believed that they were not at any risk of developing UC (Figure 4). Being 25 years and below in age (COR = 3.14, CI = 1.75–5.66, *p* < 0.001), of Yoruba ethnicity (COR = 2.00, CI = 1.05–3.63, *p* = 0.034), a Muslim (COR = 2.31, CI = 1.33–4.05, *p* = 0.003), knowing someone with UC (COR = 4.56, CI = 2.25–9.25, *p* <0.001), prior discussion with a doctor about uterine cancer (COR = 3.86, CI = 2.19–6.82, *p* < 0.001) and having good knowledge of UC (COR = 7.40, CI = 4.10–13.36, *p* < 0.001) were significantly associated with increased odds of the perception of being at risk of having UC. Being 25 years and below in age (AOR = 2.49, CI = 1.20–5.17, *p* = 0.014), being a Muslim (AOR = 3.08, CI = 1.58–5.99, *p* = 0.001), knowing someone with UC (AOR = 3.11, CI = 1.27–7.57, *p* = 0.013) and having good knowledge of UC (COR = 5.88, CI = 2.80–12.35, *p* < 0.001) were the independent factors that significantly predicted the perception of being at risk of developing UC (Table 8).

## Discussion

In the absence of a recommended screening strategy for UC, and the increasing prevalence of the disease, there is an urgent need to invest in public health interventions that would encourage early presentation of suspicious symptoms for prompt investigation, early diagnosis and treatment to improve health outcomes. A major step towards achieving this goal is to improve public knowledge about the disease, especially among at-risk populations. Our study assessed the knowledge of UC’s symptoms, risk factors, risk-reducing health measures, their perceived risk of developing UC and the factors influencing it among women in Lagos, Nigeria.

We found that 25.0% of women had an overall good knowledge of the symptoms, risk factors and practices that reduce the risk of UC, while the majority (39.3%) and 35.7% had no knowledge and poor knowledge, respectively. Specifically, 34.9%, 27.4% and 39.3% had good knowledge of the symptoms, risk factors and risk-reducing measures of UC, respectively. The majority (88.8%) believed they were not at risk of developing UC; while 11.2% perceived that they may be at risk of developing UC. Age of 25 years and below, having at least a secondary education, being unmarried, being a Christian, knowing someone with UC and prior discussion with a doctor about UC were independent factors that significantly predicted good knowledge of UC. On the other hand, being 25 years and below in age, being a Muslim, knowing someone with UC and having good knowledge of UC were the significant independent factors that positively influenced self-perception of the risk of developing UC.

The level of awareness of UC among the study participants was 58.4%, similar to the 57.0% [20] and 51.5% [18] reported in another state in Nigeria and Germany, respectively, lower than that in Sri Lanka (83.6%) [30] and higher than the 6% reported in a rural setting in Australia [19] and the zero level of awareness report among elderly women in Egypt [21]. Our finding is consistent with the reported low public awareness level of UC, even in high-income countries with a high disease burden [5]. The low awareness is believed to be responsible for the disproportionate poor funding allocation from donor agencies for UC research work and ultimately the low research output in UC compared to cervical and ovarian cancer. It is, therefore, not surprising that bridging the knowledge gaps about UC awareness was one of the top ten unanswered research questions in UC identified by a wide variety of stakeholders [15].

The knowledge about specific aspects of UC such as the symptoms, risk factors and risk-reducing behaviours was abysmally low among women in the study with the majority of the respondents having no knowledge of any of these aspects. Only 34.9% had good knowledge of the symptoms of UC and this poor level of knowledge is comparable to findings in the UK where only 24% of women could identify 2 or more symptoms [16]. The most commonly identified symptom of UC was vaginal bleeding after menopause which is similar to findings in another study in Sri Lanka [30] and comparable to the symptoms reported by women with UC [11]. However, the proportion of women who recognised this symptom as that of UC was low (27.4%) compared to the 54.9% and 62.7% reported in Colombo, Sri Lanka [30] and Minnesota, USA [31], respectively. The low recognition rate of this symptom and other symptoms of UC may be in keeping with the reported normalisation of UC symptoms by women which may lead to delayed presentation and prevent early diagnosis.

Knowledge of the risk factors of UC was the lowest among the women as barely a quarter (27.4%) of the participants was adjudged to have good knowledge while a significant proportion (64.3%) had no knowledge of the risk factors. This is in tandem with findings in another study where 63% of women could not identify common risk factors for UC [19] and in the Womb Cancer Awareness Measure study where only 13% of the women could identify more than 2 unprompted risk factors for endometrial cancer [16]. The most common risk factors known by the participants were the family history of UC, advanced age, use of unopposed estrogen hormone drugs and obesity similar to what was reported by Washington *et al* [17] and Salani *et al* [24]. Obesity is a major risk factor for the development of endometrial cancer [7] and it is reported to be associated with endometrial cancer in 40%–57% of cases [32, 33]. In Lagos, Nigeria, more than 90% of EC patients had obesity [11]. Awareness of this risk factor is, therefore, critical among black women who have a higher prevalence of obesity and are at higher risk of developing UC [5, 7]. Regrettably, the awareness of obesity as a risk factor for UC is extremely low among women in our study as less than a quarter (22.7%) of women identified obesity as a risk factor for UC. This is comparable to what has been reported in several studies [16, 17, 23, 19, 30, 34, 35] where most women did not recognise obesity as a risk factor for UC. This highlights the need to improve public knowledge about UC risk factors especially the role of obesity in the development of UC to enhance appropriate risk perception and health-seeking behaviour among women.

Another critical aspect of uterine cancer awareness is the knowledge about the health measures and practices that reduce the risk of UC or enhance its early detection. This awareness can positively improve healthy lifestyle living and care-seeking behaviour among women. In line with the pattern of findings in our study, the knowledge about UC risk-reducing health measures and practices was low with approximately 60% of the participants lacking good knowledge about these practices. Almost half of the participants lacked complete knowledge about UC risk-reducing health measures and practices, similar to the complete lack of knowledge about these practices reported by El-Sayed Eldardery *et al* [21] in Egypt. This poor level of knowledge further highlights the need for an educative health awareness campaign about UC among women to enhance early detection and improve health outcomes [24]. The control of obesity being a major contributory factor to UC development is crucial in preventing UC; however, only half of the participants identified that preventing obesity reduces the risk of UC contrary to the finding in another study where more than two-thirds of the women did [24].

The consistently low level of knowledge reported about the different aspects of UC in our study reflects the generally poor understanding of UC among women in Nigeria. It is, therefore, not surprising that only a meagre quarter of the women in the study had a satisfactory comprehensive knowledge of UC. This shows that most women are not empowered with appropriate and essential information about UC that will enable them to identify early warning signs, assess their risk of developing UC and how to modify some of these factors through their behavioural and lifestyle changes.

Our study highlighted several factors that predicted women’s knowledge about UC. Young age and having at least secondary level of education significantly predicted good knowledge of UC. This partly aligns with findings reported by George *et al* [19] where education level was associated with a better understanding of UC while age did not. On the other hand, similar findings were reported in Turkey [36] and in India [37] where knowledge of gynaecological cancers and cervical cancer, respectively, were higher among young and educated women. This is probably as a result of increased access to information on UC by young and educated women through different sources especially the electronic, print and social media. The impact of knowing someone with cancer and discussion with a doctor about cancer has been shown to significantly influence or predict knowledge of cancer and its prevention in previous studies [28, 29]. Likewise, these factors significantly predicted good knowledge of UC in our study. This is not surprising as physicians and healthcare practitioners play a crucial role in health promotion and education, and their perspectives on health-related issues are respected by the clients they interact with. Similarly, the practical and experiential knowledge of cancer derived from knowing a close friend or relative with cancer also plays an impactful role in influencing knowledge and attitude towards cancer compared to mere theoretical awareness of the disease.

Risk perception is another concept that influences human participation in disease screening and prevention programs. This is the perceived likelihood or susceptibility of one developing an adverse outcome such as cancer. It is a key factor that influences cancer-related health behaviours such as participation in cancer screening programs, and prompt reporting of warning symptoms and signs to healthcare practitioners for early evaluation and early treatment of the disease [38, 39]. Unfortunately, the level of risk perception of UC among women in our study is abysmally low. Barely a tenth of the participants (11.2%) believed that they may be at risk of having UC. This low-risk perception level has significant implications for UC prevention and early detection. Women who do not perceive that they are at risk of UC are more likely to normalise warning symptoms and signs of UC, have poor healthcare-seeking behaviour and have poor health outcomes due to late presentation. For appropriate cancer risk perception, knowledge about the disease condition is crucial [40]. Good understanding UC especially its risk factors is essential for appropriate self-perception of UC risk and good health-seeking behaviour. This is consistent with the findings in our study that showed that having a good knowledge of UC significantly improved the perception of the risk of developing UC. Unfortunately, most participants lacked complete knowledge or had poor knowledge of UC, including its risk factors, which may have contributed to the low-risk perception level observed in the study. In like manner, knowing someone with UC, significantly enhanced self-perception of the risk of developing UC which is consistent with findings that showed that knowledge especially experiential knowledge plays a critical role in cancer risk perception [41]; however, this alone may not directly translate to practical attitudinal changes [42]. This is because human health behaviour is a complex interaction of biological, cognitive, emotional, motivational and interpersonal processes that is pivotal to cancer prevention, morbidity and mortality [43]. Cancer and non-communicable diseases prevention is majorly a personal responsibility that requires knowledge-based decisions derived from appropriate public health education, promotion and interventions leading to positive behavioural and lifestyle changes [44]. This concept underpins health-related behavioural theories such as the Health Belief Model theory where perceived severity, perceived susceptibility, perceived benefits, perceived barriers, cues to action and self-efficacy determine health-related behaviour [45]. Knowledge, belief, perception and attitude are central factors that influence positive behavioural changes, in addition to other social and environmental factors [44, 46]. These factors influence a woman’s lifestyle, health-seeking behaviour, interpretation and response to danger signs and symptoms of UC and early presentation for care. Unfortunately, the knowledge about UC and the perceived susceptibility to the disease among our study participants is abysmal with a high likelihood of the respondents developing a nonchalant attitude and behaviour toward early danger signs and symptoms of UC and its risk-reducing measures. This nonchalant attitude results in the normalisation of symptoms of UC, and poor health-seeking behaviour, leading to late presentation and poor health outcomes. Consequently, there is a need for appropriate and adequate information and knowledge about UC to enhance the self-efficacy and self-care ability of the woman to initiate appropriate health-related measures and behaviours that would improve her health, reduce her risk of developing UC and enhance early detection and treatment of the disease [20, 47].

### Strengths and limitations

There is a dearth of knowledge about UC and the public perception of the disease in Nigeria. To the best of our knowledge, our study is the first to evaluate women’s knowledge of UC symptoms, risk factors, risk-reducing health measures and their perceived risk of developing UC in Lagos, a cosmopolitan and metropolitan state in Nigeria. The study utilised a random sampling strategy to recruit participants from the State to improve their representativeness.

However, our study has some limitations worth considering. The study design was a cross-sectional study that only permits a snapshot evaluation of the participant’s knowledge and associated factors. There were no interventions to evaluate the impact of health education and information on participants’ knowledge. Furthermore, the survey tool did not include the complete constructs of the Health Belief Model or other behavioural models to assess the impact on health-related behavioural changes. Another limitation worth considering is the fact that our survey tool was not formally validated with statistical tests. However, the survey instrument was adapted from the tools used in previous studies and from an extensive literature search which was thereafter reviewed by experts and piloted among a convenient sample of women with similar characteristics to the study participants before it was used for the survey. Interviewers were used to collect data from the study participants and this is associated with the risk of social desirability and recall biases which may influence the responses of the participants. The strategies utilised to minimise these biases have been discussed in detail in the methodology section. Finally, our findings may not be generalisable to the wider population as the study participants were limited to women who attended healthcare facilities, specifically government-owned secondary healthcare facilities. As a result, the findings may not be a true reflection of the knowledge of UC in the general population.

## Conclusion

The knowledge about UC as it relates to its symptoms, risk factors, risk-reducing health measures and practices is very low with most women having an extremely poor overall knowledge about the disease in Lagos, Nigeria. In addition, a large proportion of women do not believe that they are at risk of developing UC. Age of 25 years and below, having at least a secondary education, being unmarried, being a Christian, knowing someone with UC and prior discussion with a doctor about UC were independent factors that significantly predicted good knowledge of UC. Similarly, being 25 years and below in age, being a Muslim, knowing someone with UC and having good knowledge of UC were the significant independent factors that positively influenced self-perception of the risk of developing UC.

There is a need by the government and stakeholders to urgently address the poor public knowledge and risk perception about UC among women through strategic public health educational interventions and campaigns bearing in mind the lack of an established screening strategy for UC. There is also a need to educate healthcare providers about UC and empower them to educate women, especially postmenopausal women about the danger signs and risks of UC. A more inclusive population-based study is also advocated to assess the knowledge and perception about UC in the general population. Furthermore, future interventional studies to assess the impact of health education and information on knowledge of UC and behavioural changes based on established behavioural theoretical frameworks would be greatly beneficial.

## List of abbreviations

ED, Endometrial cancer; HIC, High-income countries; LGA, Local Government Area; LMIC, Low- and middle-income countries; UC, Uterine cancer; UK, United Kingdom; US, United States of America.

## Conflicts of interest

The authors have no competing interests.

## Funding

None.

## Ethical approval

Ethical approval with reference number: ADM/DCST/HREC/APP/2283 and date: 8/02/2018 was obtained from the Human Research and Ethical Committee of LUTH before conducting the study. Additional approval was obtained from the Lagos State Health Service Commission and the management of the secondary health facilities before commencing the study. Verbal informed consent was obtained from the study participants after they were informed about the study before their recruitment and enrolment in the study. They were informed that participation in the study was voluntary and assured of confidentiality and protection of their identity throughout the study. The study was carried out in accordance with the Declaration of Helsinki (1964).

## Data availability

The data underlying this article will be shared upon reasonable request to the corresponding author.

## Author contributions

AAO conceptualised the study, AAO and ROS designed the study protocol and supervised the planning and execution of the study. Data collection, entry and cleaning were done by ROS, while data analysis and interpretation were done by AAO and ROS. Review of literature was carried out by AAO, ROS, OEF, SAY and FOG. AAO drafted the manuscript and all authors read and approved the final manuscript. AAO is the guarantor of the paper.

## Figures and Tables

**Table 1.** Characteristics of study participants.

Variable	Frequency (*N* = 507)	Percentage (%)	95% Confidence interval (%)
Sociodemographic characteristics			
Age in years			
20–29	193	38.1	33.7–42.2
30–39	226	44.6	40.2–48.9
40–49	57	11.2	8.7–14.0
≥50	31	6.1	3.6–9.3
Occupation			
Skilled	120	23.7	19.9–27.4
Semi-skilled	97	19.1	15.66–22.7
Unskilled	131	25.8	22.1–29.6
Unemployed	159	31.4	27.4–35.5
Highest level of education			
Tertiary	198	39.1	34.3–43.2
Secondary	164	32.3	28.4–36.5
Primary	90	17.8	14.6–21.5
None	55	10.8	8.3–14.0
Ethnicity			
Yoruba	307	60.6	56.0–64.9
Igbo	127	25.1	21.3–28.6
Hausa	31	6.1	3.9–8.3
Others*	42	8.2	5.9–11.0
Marital status			
Single	125	24.7	21.1–28.8
Married	377	74.4	70.0–77.9
Separated/ divorced	5	0.9	0.0–2.4
Religion			
Christianity	339	66.9	62.5–70.8
Islam	168	33.1	29.2–37.5
Reproductive characteristics			
Previous deliveries (Parity)			
0	195	38.5	34.3–42.6
1–2	183	36.1	32.0–40.2
3–4	111	21.9	18.5–25.6
> 4	18	3.6	2.0–5.3
Children alive			
0	217	42.8	38.3–47.1
1–2	194	38.3	33.5–42.6
>2	96	18.9	15.8–22.9
Menstrual status			
Pre-menopausal	447	88.2	85.2–90.9
Menopausal	60	11.8	9.1–14.8
Relational variables			
Knowledge of someone with uterine cancer			
Yes	44	8.7	6.3–11.2
No	463	91.3	88.8–93.7
Discussion with doctor(s) about uterine cancer			
Yes	118	23.3	19.3–27.2
No	389	76.7	72.8–80.7

* Others include Urhobo, Ijaw, Kanuri, Ibibio, Tiv, Edo

**Table 2.** Symptoms of uterine cancer identified by the respondents.

Variable	Frequency (*N* = 507)	Percentage (%)
Identified symptoms of uterine cancer^*^		
Vaginal bleeding after menopause	139	27.4
Lower abdominal pain	128	25.2
Heavy or prolonged uterine bleeding	125	24.7
Unexplained weight loss	122	24.1
Vaginal bleeding in between normal periods	120	23.7
Tiredness/ weakness	119	23.5
Abnormal vaginal discharge	118	23.3
Unexplained low blood level	98	19.3
Loss of appetite	97	19.1
Constipation	92	18.1
Abdominal swelling	91	17.9

* Multiple responses observed

**Table 3.** Risk factors of uterine cancer identified by the respondents.

Variable	Frequency (*N* = 507)	Percentage (%)
Identified risk factor of uterine cancer^*^		
Family history of uterine cancer	148	29.2
Advanced age	118	23.3
Use of unopposed estrogen hormone drugs	118	23.3
Obesity	115	22.7
Personal or family history of colon cancer	97	19.1
Late age at stopping menstrual period	94	18.5
Prolonged period of anovulation	87	17.2
Early age at first menstrual period	84	16.6
Lack of child delivery	81	16.0
Diabetes mellitus	71	14.0
Hypertension	67	13.2
Use of tamoxifen	66	13.0

* Multiple responses observed

**Table 4.** Risk-reducing health measures of uterine cancer identified by the respondents.

Variable	Frequency (*N* = 507)	Percentage (%)
Uterine cancer risk-reducing health measures^*^		
Eating healthy diets	265	52.3
Having regular check-ups with the gynecologist, especially in the presence of risk factors for uterine cancer	258	50.9
Maintaining healthy weight	255	50.3
Engaging in regular exercises and physical activities	245	48.3
Avoiding the use of estrogen-only medication	164	32.3
Using birth control pills	106	20.9
Using progesterone-containing intrauterine device	90	17.8

* Multiple responses observed

**Table 5.** Socio-demographic factors influencing knowledge of uterine cancer.

Variables	Knowledge of uterine cancer		
Age	Good knowledge (%) *N* = 127	Poor/No knowledge (%) *N* = 380	*p* value
20–29	59 (46.5)	134 (35.3)	**0.035**
30–39	46 (36.1)	180 (47.4)	
40– 49	11 (8.7)	46 (12.1)	
≥50	11 (8.7)	20 (5.3)	
Occupation			
Skilled	32 (25.2)	88 (23.2)	0.052
Semi-skilled	24 (18.9)	73 (19.2)	
Unskilled	22 (17.3)	109 (28.7%)	
Unemployed	49 (38.6)	110 (28.9)	
Education			
None	15 (11.8)	40 (10.5)	0.086
Primary	13 (10.2)	77 (20.3)	
Secondary	44 (34.6)	120 (31.6)	
Tertiary	55 (43.4)	143 (37.6)	
Ethnicity			
Hausa	9 (7.1)	22 (5.8)	0.210
Igbo	24 (18.9)	103 (27.1)	
Yoruba	80 (63.0)	227 (59.7)	
Others	14 (11.0)	28 (7.4)	
Marital status			
Single	50 (39.4)	75 (19.7)	**<0.001**
Married	77 (60.6)	300 (78.9)	
Divorced	0 (0.0)	2 (0.5)	
Separated	0 (0.0)	3 (0.8)	
Religion			**0.028**
Christianity	95 (74.8)	244 (64.2)	
Islam	32 (25.2)	136 (35.8)	

*p* value in bold prints are signficant

**Table 6.** Reproductive and relational factors influencing knowledge of uterine cancer.

Variables	Knowledge of uterine cancer		
	Good knowledge *n* = 127 (%)	Poor/No knowledge *n* = 380 (%)	*p* value
**Reproductive factors**			
No of deliveries			
0	59 (46.4)	136 (35.8)	**0.013**
1–2	33 (26.0)	150 (39.5)	
3–4	33 (26.0)	78 (20.5)	
>4	2 (1.6)	16 (4.2)	
Children alive			
0	66 (52.0)	151 (39.7)	**0.001**
1–2	31 (24.4)	163 (42.9)	
>2	30 (23.6)	66 (17.4)	
Menopausal status			
Premenopausal	115 (90.6)	332 (87.4)	0.336
Postmenopausal	12 (9.4)	48 (12.6)	
**Relational factors**			
Knowing someone with uterine cancer			
Yes	28 (22.0)	16 (4.2)	**<0.001**
No	99 (78.0)	364 (95.8)	
Discussion with a doctor about uterine cancer			
Yes	65 (51.2)	53 (13.9)	**<0.001**
No	62 (48.8)	327 (86.1)	

*p* value in bold prints are signficant

**Table 7.** Predictors of knowledge of uterine cancer.

Variables	Crude odd ratio	95% CI	*p* value	Adjusted odd ratio	95% CI	*p* value
Age						
≤25	2.92	1.83–4.66	**<0.001**	**2.55**	**1.36–4.77**	**0.003**
>25	1			1		
Occupation						
Skilled/semi-skilled	1.07	0.72–1.61	0.734	–	–	–
Unskilled/unemployed	1					
Education						
Secondary and above	1.57	1.18–2.52	**0.019**	1.67	1.06–2.91	**0.046**
Primary and below	1			1		
Ethnicity						
Yoruba	1.15	0.76–1.74	0.516	-	-	-
Others	1					
Delivery status						
Nulliparous	1.56	1.04–2.34	**0.033**	1.44	0.76–2.71	0.264
Parous	1			1		
Marital status						
Never married	2.64	1.71–4.08	**<0.001**	2.69	1.39–5.21	**0.003**
Ever married	1			1		
Religion						
Christianity	1.66	1.05–2.60	**0.029**	1.89	1.09–3.27	**0.023**
Islam	1			1		
Menstrual status						
Pre-menopausal	1.39	0.711–2.70	0.338	–	–	-
Post-menopausal	1					
Knowing someone with uterine cancer						
Yes	6.43	3.35–12.36	**<0.001**	6.62	3.12–14.01	**<0.001**
No	1			1		
Discussion about uterine cancer with a doctor						
Yes	6.47	4.11–10.18	**<0.001**	5.72	3.43–9.53	**<0.001**
No	1			1		

*p* value in bold prints are signficant

**Table 8.** Predictors of perception of risk of developing uterine cancer.

Variables	Crude odd ratio	95% CI	*p* value	Adjusted odd ratio	95% CI	*p* value
Age						
≤25	3.14	1.75–5.66	**<0.001**	**2.49**	**1.20**–**5.17**	**0.014**
>25	1			1		
Occupation						
Skilled/semi-skilled	1.23	0.71–2.14	0.460	-	-	-
Unskiled/unemployed	1					
Education						
Secondary and above	0.85	0.47–1.55	0.589	-	-	**-**
Primary and below	1					
Ethnicity						
Yoruba	2.0	1.05–3.63	**0.034**	1.32	0.66–2.65	0.434
Others	1			1		
Delivery status						
Parous	1.18	0.662–2.09	0.579	-	-	-
Nulliparous	1					
Marital status						
Ever married	1.42	0.71–2.83	0.321	-	-	**-**
Never married	1					
Religion						
Islam	2.31	1.33–4.05	**0.003**	3.08	1.58–5.99	**0.001**
Christianity	1			1		
Menstrual status						
Pre-menopausal	1.45	0.55–3.78	0.450	-	-	-
Post-menopausal	1					
Knowing someone with uterine cancer						
Yes	4.56	2.25–9.25	**<0.001**	3.11	1.27–7.57	**0.013**
No	1			1		
Discussion about uterine cancer with a doctor						
Yes	3.86	2.19–6.82	**<0.001**	1.16	0.55–2.45	0.693
No	1			1		
Knowledge of uterine cancer						
Good	7.40	4.10–13.36	**<0.001**	5.88	2.80–12.35	**<0.001**
Poor	1			1		

*p* value in bold prints are signficant

## Supplementary material

### Study questionnaire

**Please answer all questions by ticking by ticking the most appropriate answer.**

**A. Socio-demographic characteristics**

1. Age in years (as at last birthday)

2. Occupation (please be specific)

3. Highest level education: a. No formal education [ ]   b. Primary education [ ]   c. Secondary education [ ]    d. Tertiary education[ ]

4. Ethnic group: a. Yoruba [ ]   b. Igbo [ ]   c. Hausa[ ]   d. Others (specify)…

**B. Reproductive characteristics**

1. Total number of pregnancies delivered (specify)

2. Total number of children alive (specify)

3. Marital status: a. Single [ ]   b. Married [ ]   c. Separated [ ] d. Divorced [ ]   f. Widowed [ ]    g. Others (please specify)…

4. What religion do you practice? a. Christainity [ ] b. Islam [ ] c. Others (specify)…

5. Are you still seeing your menstrual period? a. Yes [ ] b. No [ ]

6. If no, for how long have you stopped seeing it? (please specify)…

**C. Awareness and source of knowledge about uterine cancer**

1. Have you heard of uterine cancer before? a. Yes [ ]   b. No [ ]

2. What is/are your source(s) of knowledge / information about uterine cancer?

**(You may tick more than one option as appropriate)**

a. Radio/Television [ ]   b. Internet and social media [ ]   c. Seminars/workshops [ ]

d. Newspapers/Magazines/Books [ ]   e. Doctors/Nurses [ ]   f. Friends/relatives [ ]

h. Others (please specify)..

**D. Knowledge of symptoms of uterine cancer**

**NB:** Please answer all questions and tick the appropriate answer.

The following are symptoms of uterine cancers:

**Table:**

	Yes	No	I don’t know
1. Heavier or longer menses than usual			
2. Vaginal bleeding after menopause			
3. Vaginal bleeding in between normal periods			
4. Abnormal vaginal discharge			
5. Abdominal swelling			
6. Lower abdominal pain			
7. Unexplained weight loss			
8. Pain during sexual intercourse			
9. Loss of appetite			
10. Constipation			
11. Tiredness / Weakness			
12. Unexplained low blood level			

**E. Risk factors for uterine cancer**

Please answer all questions and tick the appropriate answer.

The following are risk factors for having cancer of the uterus.

**Table:**

	Yes	No	I don’t know
1. Old age			
2. Obesity			
3. Childlessness			
4. Prolonged period of anovulation/ infertility			
5. Family history of uterine cancer			
6. Personal or family history of colon cancer.			
7. Early age at first menstrual period			
8. Late age at stopping menses [menopause]			
9. Use of unopposed estrogen hormone replacement drugs			
10. Use of tamoxifen drug			
11. Hypertension			
12. Diabetes Mellitus			

**F. Preventive health behaviours and practices**

Please answer all the question and tick the appropriate answer

The following are health behaviors that may reduce the risk of uterine cancers:

**Table:**

	Yes	No	I don’t know
1. Having regular check-ups with the Gynaecologist especially with the presence of risk factors for uterine cancer			
2. Eating healthy diets			
3. Maintaining healthy weight, i.e., avoiding obesity			
4. Being physically active and doing regular exercise			
5. Avoiding use of estrogen only medication			
6. Using progesterone containing intrauterine device			
7. Using birth control pills			

**G. Perception of risk**

1. Do you think you are at risk of having uterine cancer? a. Yes [ ] b. No [ ] c. I don’t know [ ]

**H. Relational factors**

1. Has any doctor/nurse talked to you about uterine cancer before? a. Yes [ ] b. No [ ]

2. Do know someone who has uterine cancer? a. Yes [ ] b. No [ ]
